# Comparative physiological and transcriptome analysis in cultivated and wild sugarcane species in response to hydrogen peroxide-induced oxidative stress

**DOI:** 10.1186/s12864-023-09218-3

**Published:** 2023-03-27

**Authors:** R. Manimekalai, A Selvi, Jini Narayanan, Ram Vannish, R. Shalini, S Gayathri, V.P Rabisha

**Affiliations:** Crop Improvement Division, Sugarcane Breeding Institute, Indian Council of Agricultural Research (ICAR), Coimbatore, Tamil Nadu 641 007 India

**Keywords:** *Erianthus* sp, *Saccharum* sp, sugarcane transcriptome, *NAC* genes

## Abstract

**Background:**

Sugarcane is an important energy crop grown worldwide,supplementing various renewable energy sources. Cultivated and wild sugarcane species respond differently to biotic and abiotic stresses. Generally, wild species are tolerant to various abiotic stresses. In the present study, the physiological and molecular responses of cultivated and wild sugarcane species to oxidative stress at the transcriptional levels were compared. Transcriptional responses were determined using RNAseq. The representative RNA-seq transcript values were validated by reverse transcriptase quantitative polymerase chain reaction (RT-qPCR) and confirmed through physiological responses.

**Results:**

Oxidative stress causes leaf-rolling and -tip drying in cultivated sugarcane, but the wild species are tolerant. Higher chlorophyll fluorescence was observed in the wild species than that in the cultivated varieties under stress. Wild species can maintain a higher chlorophyll stability index than the cultivated species, which was confirmed by the lower transcripts of the chlorophyllase gene in the wild species than that in the cultivated variety. Transcription factor genes (*NAC*, *MYB*, and *WRKY*) were markedly expressed in response to oxidative stress, revealing their involvement in stress tolerance. The analysis revealed synchronized expression of acetyl-transferase, histone2A, cellulose synthase, and secondary cell wall biosynthetic genes in the wild species. The validation of selected genes and 15 NAC transcription factors using RT-qPCR revealed that their expression profiles were strongly correlated with RNA-seq. To the best of our knowledge, this is the first report on the oxidative stress response in cultivated and wild sugarcane species.

**Conclusion:**

Physiological and biochemical changes in response to oxidative stress markedly differ between cultivated and wild sugarcane species. The differentially expressed stress-responsive genes are grouped intothe response to oxidative stress, heme-binding, peroxidase activity, and metal ion binding categories. Chlorophyll maintenance is a stress tolerance response enhanced by the differential regulation of the chlorophyllase gene.There is a considerable difference in the chlorophyll stability index between wild and cultivated varieties. We observed a substantial regulation of secondary wall biosynthesis genes in the wild species compared with that in the cultivated variety, suggesting differences in stress tolerance mechanisms.

**Supplementary Information:**

The online version contains supplementary material available at 10.1186/s12864-023-09218-3.

## Background

Sugarcane is an important energy crop cultivated in tropical and subtropical regions globally. Sugarcane is a significant component of bioenergy crops in many tropical and subtropical economies [1]. Approximately 100 countries produce sugarcane covering22 million ha (FAOSTAT, 2008). Increasing sugarcane productivity is essential for producing renewable energy sources and food sustainability.

Cultivated sugarcane is a complex hybrid of several species and subspecies. The majority of the commercial sugarcane varieties in India have approximately70–80%, 10–20%, and 8–13% of their chromosomes derived from *Saccharum officinarum*, *Saccharum spontaneum*, and interspecific recombination, respectively. A draft genome sequence assembly of the commercial sugarcane cultivar SP80-3280 [2] and a reference sequence corresponding to the gene-rich regions of the primary (monoploid) sugarcane genome (R 570) of 382 Mb [3] are currently available. Sorghum (*Sorghum bicolor*)is an excellent alternative reference genome for the comparative analysis and annotation of genes. Comparative mapping between sugarcane and sorghum revealed a 95.2% sequence identity in the coding region**.**

Oxidative stress is an important factor in various abiotic stresses such as high temperature, drought, salinity, light, flooding, and heavy metals [4]. Oxidative stress occurs due to an imbalance between the production of reactive oxygen species (ROS) and the antioxidant defense system in plant cells, leading to physiological and metabolic changes. Plants have evolved various molecular mechanisms to detect rapid changesand adapt. Depending on the dosage, H_2_O_2 _induces multiple responses in the cell. A high dosage leads to hypersensitive cell death [5–8], whereas a low dosage blocks cell cycle progression [9] which functions as a developmental signal for differentiation in the secondary cell wall [10].

Wild sugarcane species, such as *S.spontaneum* and *Erianthus* sp. tolerate several abiotic stresses and are sources of the tolerance genes in cultivated sugarcane through introgressive breeding. However, repeated backcrossing and selection for high yield have resulted in a narrow gene pool in the cultivated varieties; hence, the need to identify the resistant genes in the wild species foruse in breeding programs to develop stress-tolerant cultivated varieties. Several reports explained the use of hydrogen peroxide simulating oxidative stress in plants [11, 12]. Vijayalakshmi [12] used varying concentrations of H_2_O_2_ (0.05, 0.1, 0.15, 0.2 mM) to induce oxidative stress in rice.

Gene expression profiling through next-generation sequencing (NGS) has become an important tool for investigating how organisms respond to environmental changes. Understanding these reprogramming events is essential for gene discovery and introgression breeding programs aiming to enhance tolerance to multiple abiotic stresses in cultivated sugarcane. Several reports are available on the global gene expression profiles of sugarcane in response to drought [13], salinity and osmotic [14] and cold stresses [15]. In our previous study [16], we reported the expression profiles of several genes involved in oxidative stress tolerance in sugarcane. The present study focused on comparative oxidative stress responses in cultivated and stress-tolerant sugarcane wild species (*Erianthus* sp.and *S.spontaneum*) to identify oxidative stress-responsive genes using transcriptomics. The present study provides an understanding of the molecular pathways of global gene expressions involved in abiotic stress tolerance, enabling the selection of sugarcane varieties adapted to marginal environments.

## Results

### Morphological and physiological response of plants under oxidative stress

#### The cultivated and wild sugarcane species exhibited varied responses to oxidative stress

**Fig. 1:**
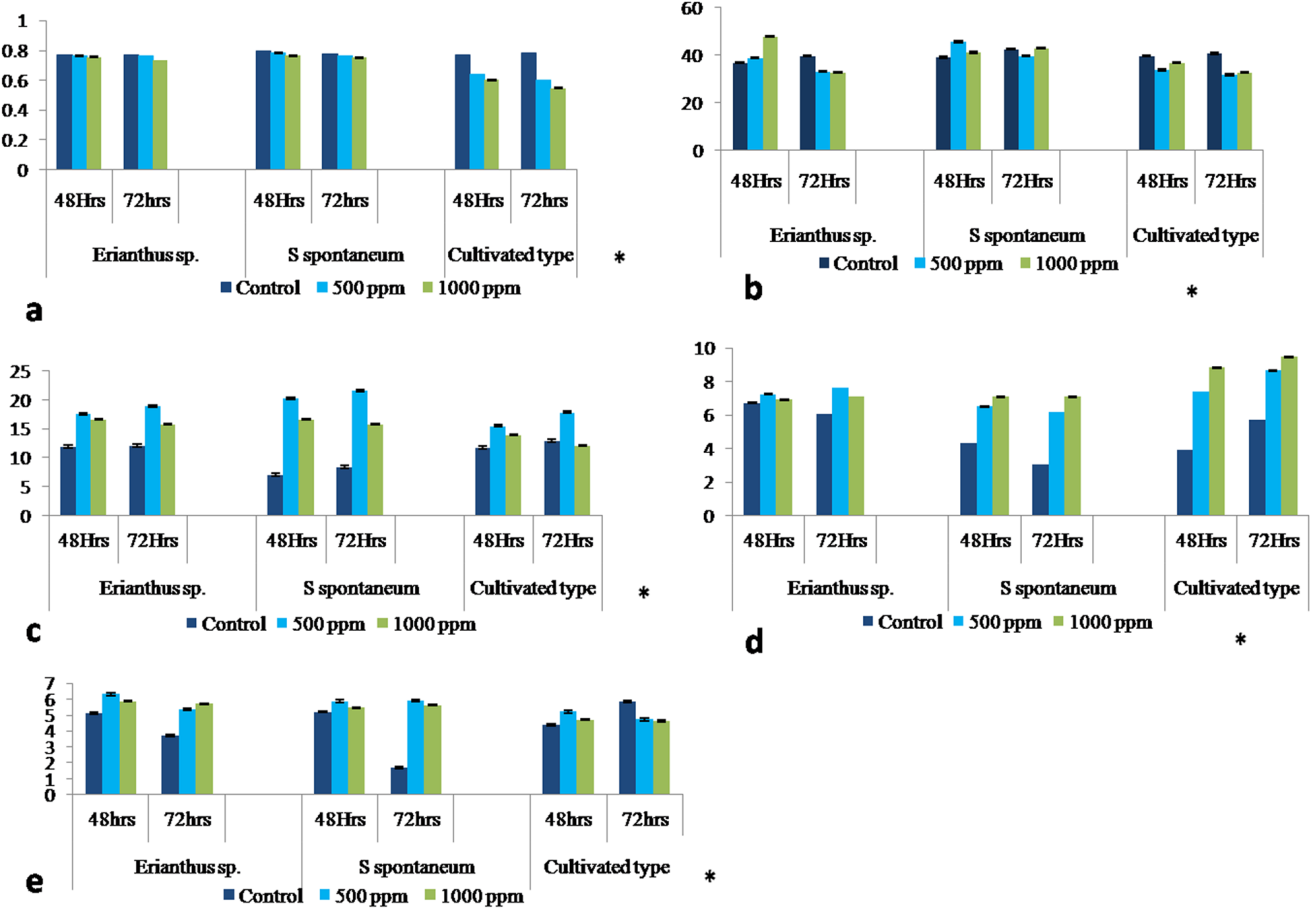
Physiological response of the plants to hydrogen peroxide. **a**. Chlorophyll fluorescence; Y axis Fv/Fm ratio. **b**. Hydrogen peroxidase levels; Y axis—POX activity—µg/g/min. **c**. Super oxide dismutase levels. Y axis—SOD activity—µg/g/min. **d**. Lipid peroxidation levels;Y axis—LPO (nmol/MDA/g.fr.wt.) **e**. Proline content; Y axis—mg/g of fr.wt**.** Statistical significance among genotypesare labeled with *

**Fig. 2:**
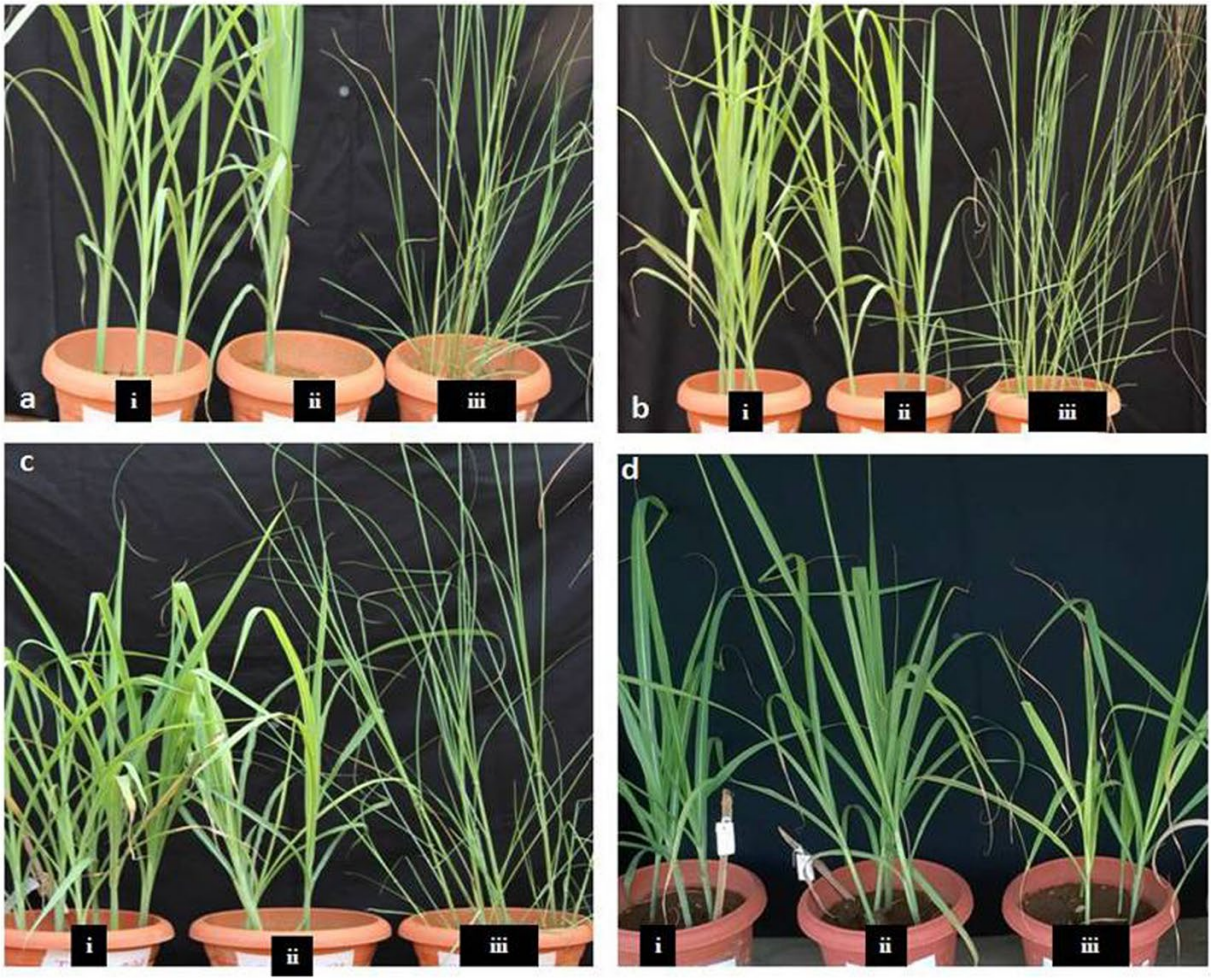
The phenotype of sugarcane and wild species under oxidative stress **a**. Control:i and ii. *Erianthus* sp; iii *S. Spontaneum ***b**. 500 ppm: i and ii. *Erianthus* sp; iii *S. Spontaneum ***c**.1,000 ppm: i and ii. *Erianthus* sp; iii *S. Spontaneum ***d**. Cultivated variety: i. Control; ii. 500 ppm; iii. 1,000 ppm. Leaf rolling and yellowing appeared in plants sprayed with 1,000 ppm hydrogen peroxide in the cultivated variety

*Erianthus* sp*.* had an average of 27.1% chlorophyll fluorescence (CF) and *S. spontaneum* had a 23.7% increase in chlorophyll stability index (CSI) over the cultivated variety under stress conditions (500 ppm). (Fig. 1a). There were substantial differences in CSI and CF between different genotypes and H_2_O_2_ concentrations (Table 1). *S. spontaneum* exhibited considerable increases in H_2_O_2_ and superoxide dismutase (SOD) activity under stress conditions (Fig. 1b and c). Both wild species had low peroxidation values (Fig. 1d). There were considerable differences in SOD, peroxidase (POX), and lipid peroxidation (LP) concentrations between the different genotypes and H_2_O_2_ concentrations. *S. spontaneum* exhibited high proline concentration under stress conditions. A 22.5% average increase in proline concentration was observed in wild species (500 ppm 72 h treatment) compared with that in the cultivated variety (Fig. 1e) (Table 1). Overall, the wild species (IMP-564 and SES-90) and Co 86,032 cultivated variety exhibited considerable differences in physiological and biochemical parameters. There were no visible morphological changes between *Erianthus* sp.and *S. spontaneum* compared with those of the control, at 500 and 1,000 ppm, but leaf-lamina drying and leaf-rolling were observed at 500 and 1,000 ppm treatments (Fig. 2).

**Table 1:** ANOVA for physiological and biochemical parameters

Factor	dF	F ratio and probability					
		**SOD**	**POX**	**Proline**	**CSI**	**LPO**	**CF**
*Genotypes* (V)	2	17.96**	2.16**	3.47*	19.23**	5.3**	4.973**
H_2_O_2_ spray(S)	2	5.56***	4.12*	1.8^ ns^	3.12*	0.97^ ns^	2.100^ ns^
Time (T)	1	2.92^ ns^	0.08^ ns^	0.02^ ns^	3.28*	3.96*	2.946**
V x S	4	1.75^ ns^	1.31^ ns^	1.82^ ns^	4.94*	2.34*	25.28**
V x T	2	3.28*	5.2**	2.98*	0.003^ ns^	3.19*	1.114^ ns^
S x T	2	1.55^ ns^	1.6^ ns^	0.623^ ns^	0.17^ ns^	1.23^ ns^	1.17^ ns^

ANOVA shows three genotypes (Varieties-V) effects with physiological parameters during different time interval (T, 48 and 72 h) with different concentration (Spray-S, 500 and 1000 ppm) of H_2_O_2._ Note: Levels of significance: ***, **, * and ns indicate significant difference at *p* < 0.001, *p* < 0.01, *p* < 0.05 and non- significant respectively

#### De novo assembly of transcripts and annotation of expressed genes

**Fig. 3:**
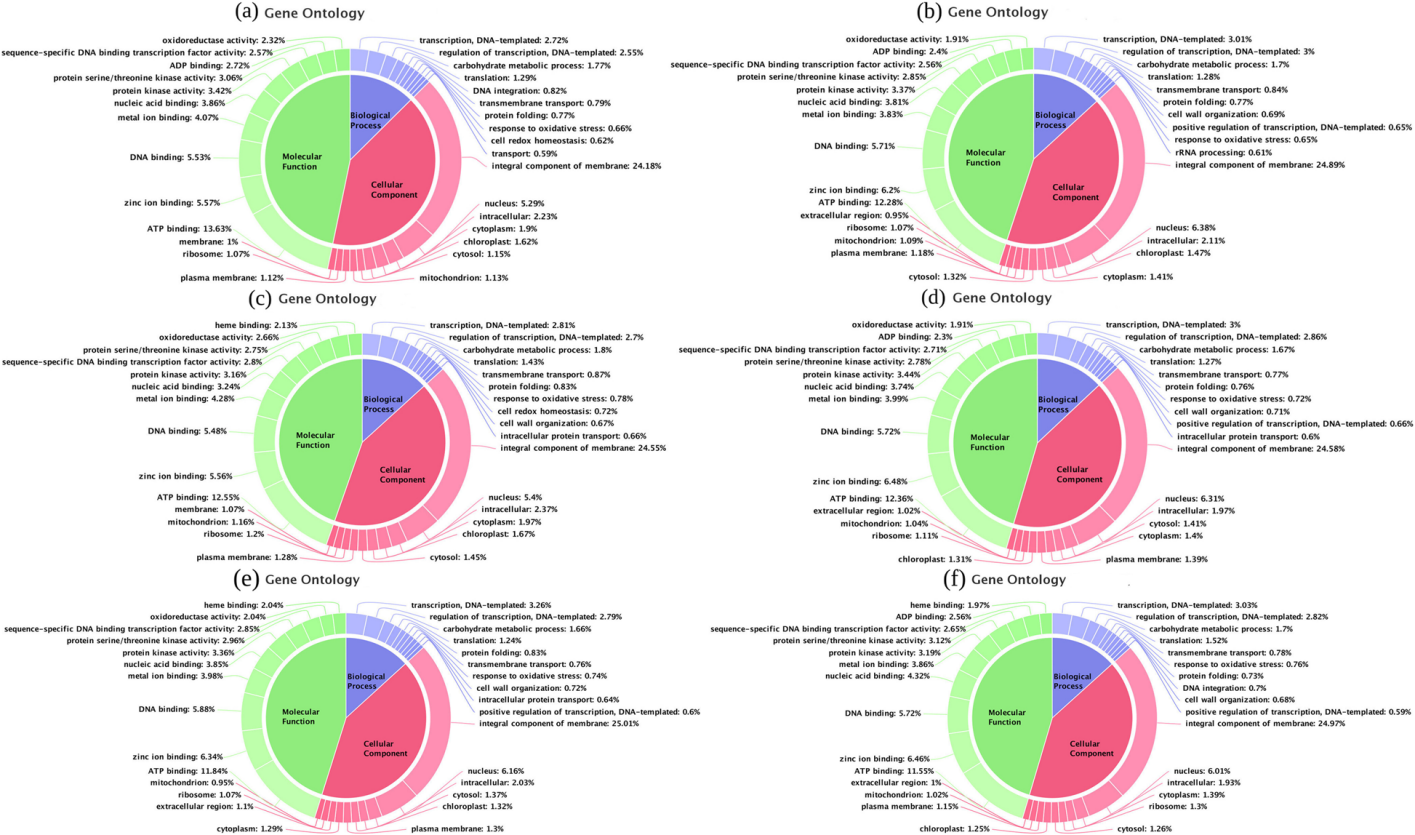
Functional characterizationof hydrogen peroxide induced gene transcripts. The pie chart shows the Gene Ontology terms (molecular function, biological process, and cellular component) of sugarcane and wild species according to the best search results based on log2 Fold Change of > 2, *P* value of ≤ 0.05. **a** Control *Erianthus* sp*.,***b** stressed *Erianthus* sp*.,***c** control *S.spontaneum,***d** stressed *S.spontaneum,***e** control cultivated variety, and (**f**) stressed cultivated variety. Pie charts were obtained using the Blast2GO software

RNA samples from *Erianthus* sp., *S. spontaneum*, and the cultivated variety were sequenced using the Illumina NextSeq500 platform and generated ~ 121, ~ 120, and ~ 117 million reads (150 bases) with GC bases ranging from 55 to 57%. The N50 was 1,111 bp for each sample. Contigs were joined and assembled into unigenes (Table 2). The total number of assembled unigenes 3,07,358 out of which 2,23,896 were annotated based on sorghum genome (Sb) (TableS1) and sugarcane **(**varietyR570 / SP80-3280) as the reference (Table S2). Gene Ontology (GO) categorization of *Erianthus* sp. (Fig. 3a and b), *S. spontaneum* (Fig. 3c and d), and cultivated variety (Fig. 3e and f) exhibited that themajority of gene transcriptswere in the “molecular function” (46, 45, and 45%, respectively) and “cellular component” (41, 42, and 41%, respectively) and the least number of transcripts were in the “biological process” (12.9, 13.4, and 13.7%, respectively). There were moregene transcripts under the molecular function and biological process categories for cultivated varieties than those for the wild species. The GO for *Saccharum* sp., *Erianthus* sp. and cultivated variety is provided in Table S3.

**Table 2:** Annotation summary of transcripts of sugarcane and wild species

Summary	Set 1 *Erianthus arundinaceus*		Set 2 *Saccharum spontaneum*		Set 3 Co 86032	
	**Control**	**Treated**	**Control**	**Treated**	**Control**	**Treated**
Total Transcripts	64046	43311	52851	39549	52118	55483
Total Annotated transcripts (UniProt)	42204	34512	37919	32858	39696	36707
Total Unannotated transcripts	21842	8799	14932	6691	12422	18776

### Differentially expressed gene transcripts

We used the DESeq tool to identify differentially expressed genes between the control and oxidative stress conditions and filtered them based on their fold changes (> 2) and *p*-values (*p* < 0.05). We identified differentially expressed gene transcripts between species under oxidative stress conditions. There were 25, 51, and 30 upregulated and 105, 45, and 54 down regulated (farnesol dehydrogenase) gene transcripts in the *Erianthus* sp.*; S. spontaneum;* and cultivated variety, respectively. *S. spontaneum* exhibited relatively high expression values for the endo glucanasegene (FPKM [18,646]), glycan biosynthesis, lipid metabolism, and starch and sucrose metabolism. Few of the upregulated genes are ferredoxin nitrate reductase, cellulose synthase and peroxisome related genes. The complete lists of annotated differential gene expressions (DGEs) and their FPKM are provided in Table S4. The gene transcripts related to photosynthetic carbon fixation exhibited lower expression levels in the cultivated than that in wild species. Many differentially expressed transcripts were annotated as hypothetical genes; 94, 31, and 30 upregulated and 291, 139, and 297 down-regulated transcripts in *Erianthus* sp*.*; *S. spontaneum*; and cultivated variety, respectively, may be novel in the *Saccharum* complex (Table S5).

### Differentially expressed transcription factor genes

**Fig. 4:**
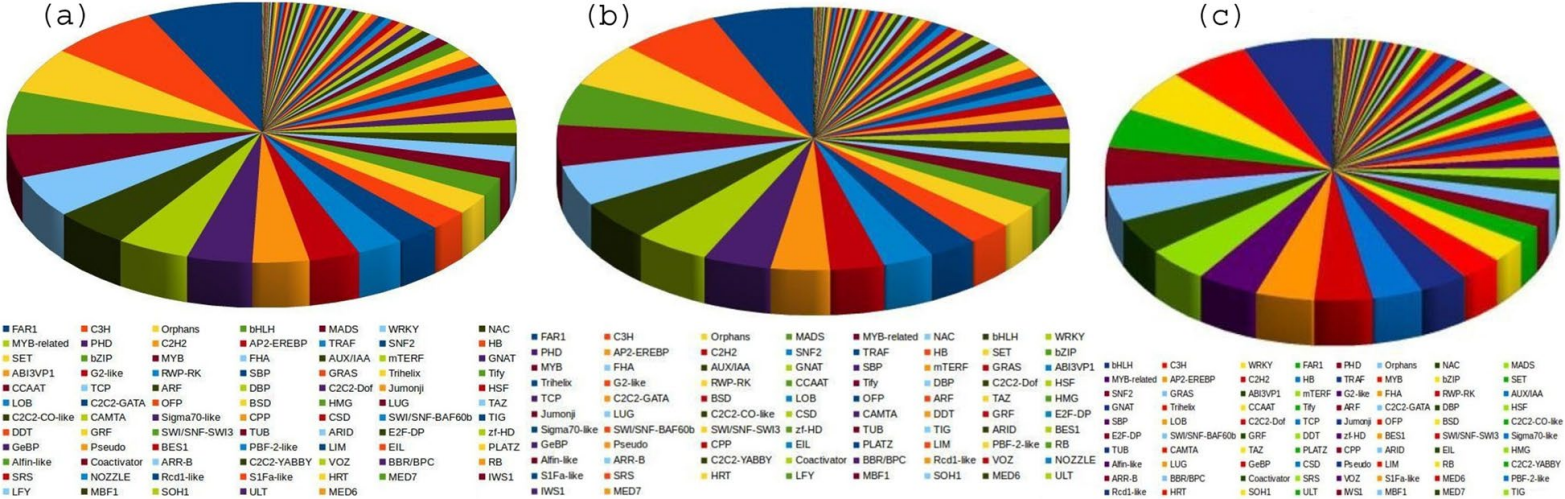
Distribution of transcription factors (TFs) of control and stressed sugarcane and its species. 4**a** represents the control and stressed *Erianthus* sp.; 4**b** represents the control and stressed *S. spontaneum;* and 4**c** represents the control and stressed cultivated varieties. Pie chart constructed based on percent identity, alignment length, E-value, TF_Family, and TF_Domain

**Fig. 5:**
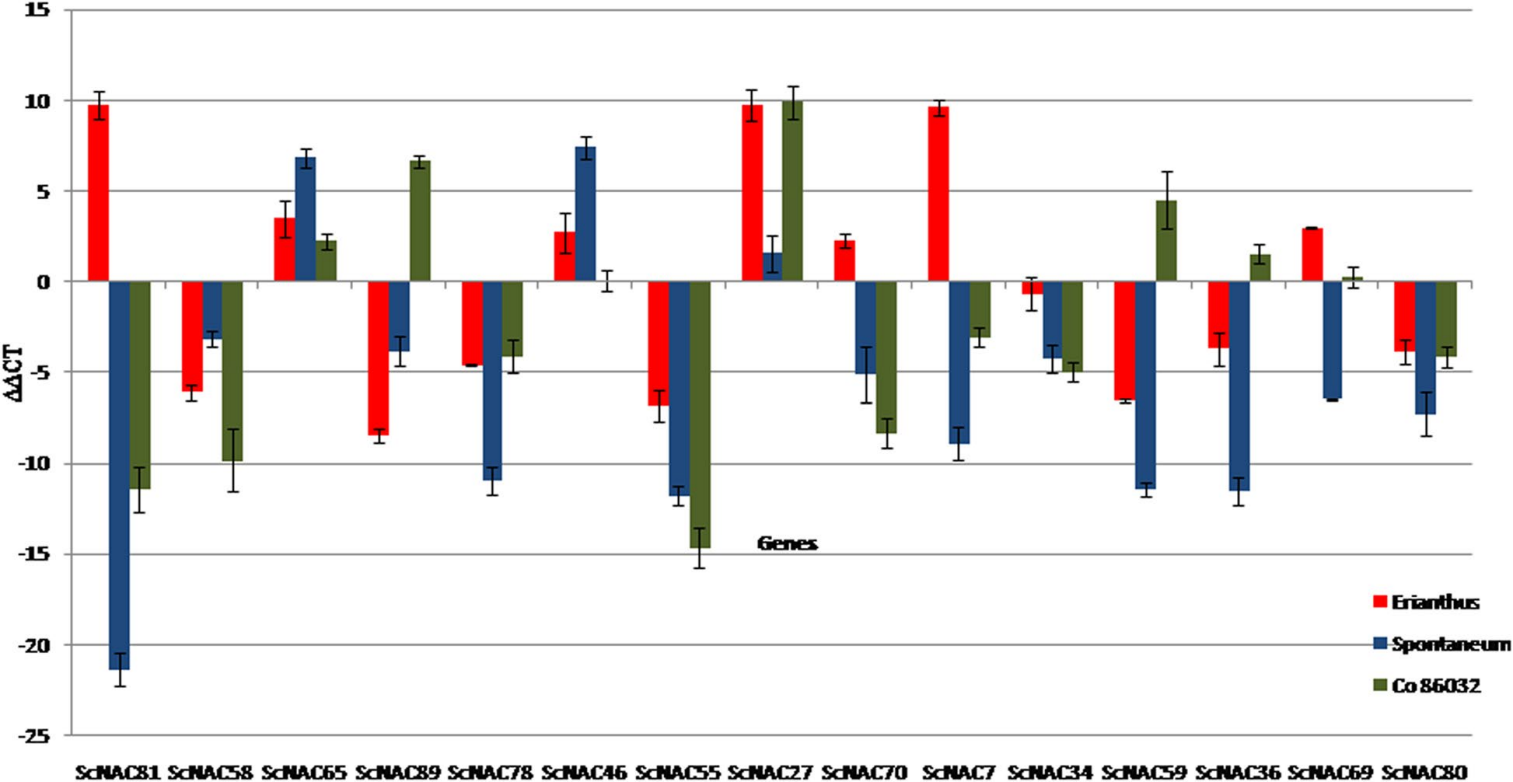
RT – PCR based validation of expression profiles of NAC TFs. The red bar represents *Erianthus sp*.*,* yellow bar represents *S spontaneum,* and blue bar represents the cultivated variety. Error bars indicate the columns. Quantification was done with Delta-Delta Ct normalization method. ddCt = dCt_(Treated)_—dCt_(Control)_. Ct is on a log scale, base 2

In the present study, we explored important biotic and abiotic stress-responsive transcription factor families in sugarcane and other species. An average of 736 C3H (Zn finger domain) and 650 far-red responsive 1 (FAR1) transcripts were identified. FAR are positive regulators of chlorophyll biosynthesis via activating the expression of the *HEMB1* gene [17]. FAR1, C3H, MADS, bHLH, and NAC had high percentages in the control *Erianthus* sp. and low percentages in stressed *S. Spontaneum*** (**Table 3)**.** Similarly, high and low percentages of orphans were observed in non-stressed *Erianthus* sp. and the cultivated variety, respectively. A high and low C2H2 TF percentage was observed in the stressed cultivated variety and stressed *S.spontaneum,* respectively. Other TFs, such as MYB-related and AP2- ethylene-responsive gene (*EREBP*), were high in then on-stressed cultivated variety and low in the stressed *Erianthus* sp. and non-stressed *S.spontaneum*. Among the other large TF families, NAC, MYB, and WRKY putative TFs were discussed considering FPKM. An average of 446 MYB, 434 WRKY, and 433 NAC transcripts were differentially expressed. The distribution of differentially regulated transcription factors (TFs) is illustrated in Fig. 4, and the details of the differentially expressed TF genes are provided in Table S6.The expression levels of three upregulated and nine downregulated (> twofold change in up- or down-regulated under oxidative stress conditions) NAC transcripts were validated using RT-qPCR. Table 4]). The comparative expression profiles of NAC genes (Fig. 5) exhibited ScNAC59 and ScNAC80 down regulation in cultivated varieties and up-regulation in wild species. Contrastingly, the ScNAC65 and ScNAC46 transcripts were down-regulated in wild species and upregulated in cultivated varieties. The comparison between the NGS values and qPCR-validated profiles for the cultivated and wild sugarcane species is provided in Table S7.

**Table 3:** Distribution of differentially expressed transcription factors in sugarcane and related species

Family	Count					
	**Control *Erianthus arundinaceus***	**Stressed *Erianthus arundinaceus***	**Control *Saccharum spontaneum***	**Stressed *Saccharum spontaneum***	**Control Co 86032**	**Stressed Co 86032**
FAR1	765	571	571	524	583	643
C3H	834	703	703	663	737	781
Orphans	592	358	358	356	348	445
MADS	570	430	430	408	541	555
bHLH	550	466	466	405	507	502
WRKY	546	391	391	404	388	487
NAC	486	403	403	395	439	474
MYB-related	485	381	381	403	490	541
PHD	459	418	418	427	467	487
C2H2	358	320	320	308	347	363
AP2-EREBP	331	276	276	271	351	310

**Table 4:** Details of NAC selected for the validation of their expression profiles

**Gene Name**	**Primer Name**	**Primer Sequence(5’-3’)**	**AnnealingTemp(°C)**	**Product Size(bp)**	**Unigenes**
ScNAC 78	s1_NAC1 F	ACGGGGTACAACTGAACTGC	60	166	Sample_1_c26124_g1_i2
	s1_NAC1 R	CCAACTGTTGCAGTGCCTAA	59.9		
ScNAC46	s1_NAC3 F	GGCAGCTTCAGTTTCTTGCT	59.8	182	Sample_1_c28778_g2_i1
	s1_NAC3 R	AGCGTGATGAACAACAGCAG	60.1		
ScNAC55	s1_NAC5 F	AGCAAGCAACAAGGGAAGAA	60	201	Sample_1_c28922_g1_i2
	s1_NAC5 R	AGGACAAGGTCACCAGGTTG	60		
ScNAc89	s1_NAC6 F	CTGGATGTCGTCGTAGCTGA	60	176	Sample_1_c29565_g1_i2
	s1_NAC6 R	AGTCGGAGATCGTGGACAAC	60.1		
ScNAC36	s2_NAC2 F	GCGGTCTGGACGGATAATAA	59.9	214	Sample_2_c29193_g1_i2
	s2_NAC2 R	ACTGAAGCCTGAACGACGAT	59.9		
ScNAC81	s2_NAC4 F	CACAAACTCATCCGGCTACA	59.7	195	Sample_2_c22709_g2_i1
	s2_NAC4 R	GAGCTGGAAGAACGACGAAC	60		
ScNAC58	s3_NAC2 F	TGTTTCCACATTGCTGGTGT	60	150	Sample_3_c42536_g1_i3
	s3_NAC2 R	CCTTTTGGAGAGTGCTCTGG	60		
ScNAC27	s3_NAC3 F	AGTCGGAGATCGTGGACAAC	60.1	170	Sample_3_c41194_g1_i8
	s3_NAC3 R	CTGGATGTCGTCGTAGCTGA	60		
ScNAC69	s5_NAC1 F	TAATCCGGCCGGTAGTAGTG	60	158	Sample_5_c29179_g1_i1
	s5_NAC1 R	TTCTCCCTCTTGCATTGCTT	60		
ScNAC34	s5_NAC4 F	GGTGAACCAGTCGTTGTCCT	60	228	Sample_5_c29442_g1_i1
	s5_NAC4 R	AAGGTGAAGGTGGAGAAGCA	59.8		
ScNAC70	s5_NAC6 F	CTTCGCAAAGAGGTGGAAAG	60	209	Sample_5_c16801_g1_i1
	s5_NAC6 R	CTTTGGAGTTTTCGGACCAA	60.1		
ScNAC7	s5_NAC7 F	CCGGATCGATTAGTTGGTTG	60.3	228	Sample_5_c34885_g1_i2
	s5_NAC7 R	CCAACCTGGAGCTACGAGAG	60		
ScNAC59	s5_NAC8 F	ATAAGGTGGCCACAGACTGG	60	191	Sample_5_c11486_g1_i1
	s5_NAC8 R	GCTGTTGAGGAACAGCAATG	59.4		
ScNAC80	s6_NAC1 F	CACGACGTTGCCATTATCTG	60.1	237	Sample_6_c31836_g1_i1
	s6_NAC1 R	GAAGGAGGCAGGCATTGTAG	59.8		
ScNAC65	s6_NAC4 F	TCTGCGAAACACAGGAGATG	60	226	Sample_6_c27451_g1_i3
	s6_NAC4 R	CGCAATGCACAGGAGAGATA	60

### Gene expression validation through RT-qPCR

The expression values of four upregulated genes (dehydration responsive protein, cellulose synthase, calmodulin-binding protein, and cytochrome P450) and four down-regulated unigenes (ATP-dependent 6-phosphofructokinase (*ATP-PFK*), *POX* (EC 1.11.1.7), defensin, and ammonium transporter) were validated using RT-qPCR (Table S8). The list of genes selected for RT-qPCR validation ispresented in (Table 5), and their expression profiles are presented. The NGS data were consistent with those of the RT-qPCR for the transcripts corresponding to the dehydration-responsive protein,calmodulin-binding protein, and cellulose synthase; hence, their values are valid. Cytochrome P450 exhibited a change in expression based onthe NGS data. The POX and defensin expressions were down-regulated, confirming the reliability of the sequencing data. However, the ammonium transporter gene and *ATP-PFK* were upregulated in *Erianthus sp*. and the cultivated variety, respectively.

**Table 5:** List of representative differentially expressed transcripts and comparison of fold changes in gene expression determined by RT-qPCR, with RNA-Seq data

Gene name	RNA seq						qRT PCR					
	*Erianthus arundinaceus*		*Saccharum spontaneum*		Co 86032		*Erianthus arundinaceus*		*Saccharum spontaneum*		Co 86032	
	Log2 fold change	*P* Value	Log2 fold change	*P* Value	Log2 fold change	*P* Value	Log2 fold change	*P* Value	Log2 fold change	*P* Value	Log2 fold change	*P* Value
Dehydration responsive gene	21.64	0.00	-0.84	0.00	3.41	0.00	3.11	0.00	-1.63	0.00	3.23	0.01
Cellulose synthase (EC 2.4.1.12)	1.45	0.00	22.61	0.00	2.05	0.00	1.23	0.00	3.43	0.01	2.13	0.01
Calmodulin binding protein	0.55	0.01	17.74	0.00	2.26	0.00	1.42	0.01	2.97	0.00	1.40	0.01
Cytochrome P450	5.14	0.01	10.10	0.00	-0.68	0.00	2.21	0.01	3.56	0.00	-1.34	0.01
Posphohexokinas (EC 2.7.1.11)	-0.93	0.01	-0.83	0.00	-0.44	0.01	-1.24	0.01	-2.1	0.01	-2.65	0.00
Peroxidase (EC 1.11.1.7)	-0.78	0.00	-0.96	0.0	-0.66	0.0	-1.45	0.00	-1.09	0.00	-1.12	0.00
Defensin	5.31	0.00	-0.39	0.0	-0.91	0.0	1.54	0.00	-2.34	0.00	-3.20	0.00
Ammonium transporter	-0.87	0.00	-0.65	0.0	-0.80	0.0	-2.43	0.00	-4.60	0.01	-0.30	0.0

## Discussion

The present study reveals the physiological and molecular responsesof different species of sugarcane under oxidative stress. The entire plant molecular response was captured by transcriptome sequencing using the Illumina NextSeq500 platform. We selected two wild species (*Erianthus arundinaceus* and *S. spontaneum*) and hybrid cultivated varieties.

In our previous studies on oxidative [16], heat [18] and drought stresses [19], higher CF were observed in the wild species than that in the cultivated sugarcane under oxidative stress. The prolinecontent was 69 and 16.6% higher in *S.Spontaneum* and *Erianthus* sp., respectively, compared with those in their respective controls. LP is directly proportional to the extentof the plant damage. High H_2_O_2 _levels,accompanied by an increase in LP have been observed in young sugarcane plants during the initial growth phase under severe water stress [20]. In the present study,the wild species exhibited low peroxidation values. For example, *Erianthus* sp. had a 23% decrease in peroxidation, and *S.spontaneum *exhibited 36% lower peroxidation than that of the cultivated variety at 72 h after treatment. Several similar observations have been reported in other plants concerning abiotic stress. A lower levels oflipid peroxidation was observed in heat stress-tolerant sugarcane varieties [18, 21] and oxidative stress-tolerant sugarcane varieties [22]. Plants increase POX activity as a protective response during environmental stresses [23]. A previous study reported increased POX activity during moisture stress in sugarcane [24]. In the present study, cultivated and wild sugarcane species exhibited increased POX. In our previous study, we observed a decrease in POX at high H_2_O_2_ concentration (1,000 ppm) in cultivated varieties except *S. spontaneum*. [16]. The prolonged exposure to oxidative stress, wild species maintained higher enzyme activities under stress conditionsthan that in the cultivated variety. We observed that of the wild species, *S.spontaneum* had a better physiological and biochemical support for increased stress tolerance.

In the differential expressed transcripts under oxidative stress, there were reduced transcripts in the “cellular component” category associated with chloroplast in all species. Most transcripts were associated with chloroplast thylakoids; however, some chloroplast genes were upregulated under stress conditions in *S. spontaneum* compared with those in the cultivated variety. Under the molecular function, transcripts belonging to ATP binding, transferase activity, and heme binding had a higher expression, but the transcripts belonging to the chlorophyllase gene, alpha-amylase, and calcium ion binding had a lower expression in the cultivated variety than that in *Erianthus* sp. [25]. Based on CF and CSI physiological studies, we observed significant differences among the three genotypes (p < 0.001). However, *E. arundinaceus* had a higher CSI (27.1% increase in CF over that of the control) than that of the cultivated variety. Through RNAseq, it was observed that chlorophyllase gene transcripts were lower in wild species and higher in cultivated varieties, which may account for the degradation of chlorophyll. In the present physiological study, the CSI in the wild species was higher than that in the cultivated variety. CSI is an indicator of the maintenance of photosynthetic pigments. A previous study reported higher CSI in heat-tolerant sugarcane varieties [26]. A previous study on rice [27] indicated that chlorophyll content was reduced in susceptible varieties compared with that in tolerant varieties under salt stress.

From the most significant differentially expressed genes (DEGs), we identified gene transcripts associated with stress-related pathways. A similar study reported the upregulation of DEGs related to ethylene, jasmonic acid, oxidative burst, NBS-LRR, cell wall modification, systematic acquired resistance, and pathogen-related proteins in sugarcane plantlets [28]. Among the upregulated DEGs in *Erianthus* sp., GNAT (N-acetyl transferase) exhibited a 5-fold change. High expression of acetyl transferases increased the expression of cellulose synthase genesand conferred salt stress tolerance in plants [29]. We observed an accumulation of cellulose synthase transcripts, which increased FPKM from 239 to 6,890 in *S.spontaneum*. *S.spontaneum* exhibited upregulation of the histone H2A gene (fivefold increase) under stress conditions. The histone acetylation-associated upregulation of cell wall-related genes during salt stress was observed in maize [29]. The oxylipin biosynthetic process (GO:0,031,408) is essential for the metabolism of fatty acids in signal transduction for stomatal closure [30]. We identified upregulation of oxylipin-related genes in *Erianthus* sp. and *S.spontaneum*, but not in cultivated varieties.

Wild species and sugarcane exhibited upregulation of gene transcripts related nitrate reductase (NR) is involved in the production of NO which act as signal molecular to induce the activities of antioxidant enzymes [11]). In *S. spontaneum*, we observed the upregulation of genes related to 2-oxocarboxylic acid metabolism, andthe downregulation of genes involved in phenylpropanoid biosynthesis and lipid metabolism. In the cultivated variety, upregulated genes were related to fatty acid degradation and phenylpropanoid biosynthesis, and downregulated genes were related to peroxisomes. The upregulation of genes related to the phenylpropanoid pathway increases lignin content [31].The regulation of phenylpropanoid biosynthetic genes was downregulation in wild species and upregulation in the cultivated variety. Although the wild species have higher lignin content than that in the cultivated variety [32], stress regulates the lignin synthesis genes to favor energy metabolism over lignin synthesis in wild species. Peroxisome metabolism improves plant stress tolerance and is involvedin many important metabolic reactions, particularly abiotic stress responses [33]. Manipulation of peroxisomal scavenging systems for ROS may enhance plant fitness under environmental stress conditions [34].

Genes related to fatty acid metabolism were differentially expressed under oxidative stress. Previous reports have stated that cell membrane fatty acids are modulators of many signal transduction pathways activated by environmental stimuli [30]. The high *EREBP* expression in wild species may promotes early floral meristem identity [35] and the transition from an inflorescence meristem to a floral meristem [36].Other genes (*AP2_ARATH*) were upregulated (*AIL5*) or downregulated (*AP2*) in the wild species. *S.spontaneum* revealed a high regulation of MYB-related genes (*MY1R1_SOLTU*) involved in the regulation of drought and salt tolerance and enhanced stomatal closure in response to abscisic acid (ABA [37]) and the MYB4 gene involved in cold stress [38].

The stress-responsive *WRKY* gene expression was similar in both wild species. Seven *WRKY* genes were upregulated in the wild species but downregulated in cultivated varieties*.* Differential regulation ofgenes between wild species and cultivated variety (upregulation of *WRKY 35* and down regulation of *WRKY 46*) signals the conversion of sucrose (storage form) to reducing sugars in the cultivated variety [39]. Stress-responsive NACs  [40–43] show differential expression between wild and cultivated sugarcane species. The upregulation of *ScNAC81* and *ScNAC46* revealed the involvement of ABA-inducible leaf senescence signaling [44] and leaf senescence [45] in wild species. Over-expression of the *NAC81* (*GmNAC81*) gene in soybean is involved in stress-induced leaf senescence [46]. Down regulation of *ScNAC89* may affect plant cell division [47].The expression of *ScNAC78* revealed the induction of genes related to biosynthesis and was required for the accumulation of anthocyanins in response to high light stress [48]. *ScNAC55 *was upregulated under stress in *Erianthus* sp., and similar results were reported in *Brassica napus* L [49]. as *NAC55* modulated ROS levels and cell death. Differential regulation of *ScNAC69* (salt stress response [50], *ScNAC59* (a senescence-associated gene involvedin salt and H_2_O_2_-dependent signaling pathways [51], *ScNAC7* (involved in secondary wall biosynthesis and programmed cell death [52, 53] and *ScNAC23* (a low-temperature responsive gene [54]) revealed their involvement in stress response in sugarcane. Putative stress-associated NAC genes have previously been reported in sugarcane [55]. We observed varying expressions of NAC genes in response to oxidative stress, such as *NAC78* [56]. *NAC7*delays senescence and increases yield in maize [57]. In our previous study, NAC expression was correlated with oxidative stress tolerance [58].

RT-qPCR analysis was conducted to verify the RNA-Seq data for 8 representative and 15 NAC genes. The expression profiles of the validated genes were strongly correlated with the RNAseq expression values, confirming the reliability of the transcriptome data. Dehydration-responsive proteins, upregulated in both *Erianthus sp*. and cultivated varieties, play a significant role in plant stress tolerance [59]. The calmodulin-binding protein plays a crucial role in salt stress tolerance [60] in rice. Cytochrome P450 is upregulatedin all species involved in ROS metabolism [61]. ATP-PFK and POX expression was downregulated under oxidative stress. Down regulation of the *ATP-PFK* gene inhibits glycolysis, promotes the production of nicotinamide adenine dinucleotide phosphate, thereby protecting against oxidative stress [62]. The differences in expression between NGS and RT-qPCR may be due to the high sensitivity of RT-qPCR amplification.

## Conclusions

The present study presents the differential responses of cultivated sugarcane and wild relatives to oxidative stress through physiological and transcriptomic analyses. The present study revealed that oxidative stress induced morphological changes in the sugarcane cultivated variety and was damaged at > 500 ppm H_2_O_2_. Molecular analyses of the DEGs indicated that most genes were under theresponse to oxidative stress, heme binding, POX activity, and metal ion binding categories.The differential regulation of chlorophyllase transcripts suggests substantial differences in the CSI for stress tolerance. At the cellular level, H_2_O_2_ induces secondary wall biosynthesis in wild species compared to cultivated plants, suggesting different stress tolerance mechanisms between cultivated and wild sugarcane species. The present study discovered highly expressed stress-responsive genes in wild types. Providing a basis for the incorporation of these genes in sugarcane to develop climate-resilient crops in the future.

## Materials and methods

### Induction of oxidative stress

A pot culture experiment was designed to measure the physiological response to oxidative stress in cultivated (Co 86032) and wild sugarcane types (*S. spontaneum*, [SES-90] and *Erianthus* sp. [IK -76–91])*.* The plants were allowed to grow for 65 daysunder greenhouse conditions and treated with two different concentrations of 30% H_2_O_2_ (500 ppm and 1,000 ppm). Pots were sprayed with H_2_O_2_ (approximately 1.5 L /pot) consecutively for 3 days (48 h and 72 h) at 8:00 am Indian Standard Time, and the experiment was replicated thrice.

### Physiological response of plants under oxidative stress

Physiological changes in plants under oxidative stress were evaluated as previously described [16]. Membrane damage due to peroxidation of the lipid bilayer was evaluated by measuring the malondialdehyde content [63]. Proline,which may act as a direct scavenger of ROS or for osmolyte maintenance,was measured using the sulfosalicylic acid-acid ninhydrin method proposed by [64]. The CSI,an indicator of photosynthetic pigment maintenance, was calculated as previously described [16]. The evaluation was replicated thrice. The data on physiological parameters, such as CF, LP, and proline content and enzyme activities, such as POX and SOD, were obtained at 48 h and 72 h of H_2_O_2_ treatment. Significant differences between the treatments were tested statistically using the ANOVA (one-way/ two-way) method using JMP 9.0 software (JMP Statistical Discovery, Cary, NC, USA).

### Sample collection and total RNA extraction

Tissues (young leaves and meristem tissues) were collected in triplicate (from 65 days old plants) from plants 48 h and 72 h after spraying with H_2_O_2_, and total RNA was extracted from all the samples using TRIzol reagent (Invitrogen, Waltham, MA, USA). The isolated RNAs were treated with DNase (Promega, Madison, WI, USA) to remove residual DNA contamination. RNA samples were separatedusing 1.5% (w/v) agarose gel electrophoresis (Takara Bio, Shiga, Japan). The quality and quantity of RNA were determined using the Nanodrop spectrophotometer (Thermo Fisher Scientific, Waltham, MA, USA) and Qubit fluorometer (Thermo Fisher Scientific), and the A260/A280 ratio was 1.9, indicating the purity of RNA (devoid of DNA and protein contamination). Biological replicate samples of the isolated RNAs were pooled before RNA-Seq library preparation.

#### Library preparation and sequencing

The Illumina compact transcriptome library was constructed using the NEXTflex Rapid Directional RNA-Seq library protocol (Cat # 5138–08 [PerkinElmer Applied Genomics, Waltham, MA, USA]) according to the manufacturer’s instructions. The prepared library was quantified using Qubit (Thermo Fisher Scientific) and validated for quality by running an aliquot on TapeStation system (High Sensitivity D1000 [Agilent Technologies, Santa Clara, CA, USA]).The observed RNA integrity value for all libraries was > 9 for all RNA samples, and the transcriptome libraries were sequenced (150 bp chemistry) using an Illumina NextSeq500 instrument (Illumina, San Diego, CA, USA) at Genotypic Technologies, Bangalore, India.

#### *De-novo *assembly of sequenced transcriptome

The initial quality of paired-end raw reads obtained from the Illumina sequencer was confirmed using the FASTQC (https://www.bioinformatics.babraham.ac.uk/projects/fastqc/) tool (Illumina). Unwanted regions in the reads (adapters, low-quality reads, and ambiguous bases ‘N) were trimmed, andhigh-quality trimmed reads were obtained for further analysis. The reads from each sample were normalized and assembled *de novo *separately using Trinity [65] (K-mer25[GitHub, San Francisco, CA, USA]). Trinity-generated assemblies were clustered based on sequence similarity. Transcripts were clustered using CD-HIT (cluster database at high identity with tolerance [GitHub]) at 95% identity and query coverage to reduce the redundancy without exclusion of sequence diversity. Clustered transcripts were used for further annotation.

#### Functional annotation of transcripts

Assembled transcripts were similarly searched against the non-redundant National Center for Biotechnology Information (NCBI), Clusters of Orthologous Group [66] and Uniprot [67] databases (European Bioinformatics Institute, Cambridge, UK) using the Basic Local Alignment Search Tool (BLAST; e-value:0.00001 [PubMed, Bethesda, MD, USA]).GO terms associated with the transcripts were identified using the UniProt database. The metabolic pathway enzymes expressed in the transcriptome were identified using the Kyoto encyclopaedia of genes and genomes (KEGG) database ( [68] https://www.genome.jp/kegg/pathway.html) and the KEGG-Automatic Annotation Server tool.In the pathway analysis, a few reference organisms were selected, such as *Arabidopsis thaliana*, *Citrus sinensis*, *Fragaria vesca*, *Glycine max, Oryza sativa japonica*, *Theobroma cacao*, and *Vitis vinifera*.

### Differential gene expression

The differential expression of RNA-Seq (Bioconductor, Boston, MA, USA) was used for DGE analysis between samples [69]. Once the DGE was calculated, the genes with the expression log2-fold change greater than or less than twofold (up/downregulated) and with a *p*-value support of less than 0.01 were considered significant results.We used sugarcane (variety R570 / SP80-3280) and *S. bicolor* genomes as references for the annotation of DEGs. TF genes were classified into families, and orthologous relationships were identified using the TF genes (*WRKY*, MYB-related, *AP2-EREBP, *and others) from the PlantTFDB 5.0 (CBI, New Delhi, India [70]) comprising 320,370 sequences from 165 species of green plants and NAC TF sequences from the Grassius database [71]. All these data were used as a backend database, and TFs were identified from the transcriptome using the NCBI BLAST command and ortholog identification. The pathways were mapped for all significant transcripts in all the DGE comparison sets.

### RT-qPCR validation of DEGs

Differentially expressed transcripts between the three samples identified by RNA-Seq were confirmed by RT-qPCR to validate gene expression. Fifteen NAC genes (Table 4) and other representative DEGs (Table 5) were used. cDNA was prepared using a cDNA reverse transcription kit (Thermo Fisher). Forward and reverse primers were designed for these genes, and the elongation factor 2 gene was used as the reference gene. Three biological replicates were used for each sample. Among the identified NAC TFs, 15 were validated by RT-qPCR. The PrimeScript™ 1st strand cDNA synthesis kit (Takara Bio) was used for cDNA conversion. The 25S rRNA gene was used as a reference for the expression analysis of NAC genes. All RT-qPCR experiments were performed using the Rotor-GeneQ Qiagen Real-Time PCR system (Qiagen, Hilden, Germany) and QuantiNova™ SYBR Green PCR Kit(Qiagen). Relative quantification was performed using thedelta-delta Ct normalization method [72] and log_2_fold change (Log_2_FC).

## Supplementary Information

**Additional file 1. Supplementary file. I: **Summary of Annotation for DEGs of *Erianthus arundinaceus, Saccharum spontaneum *and cultivated variety.

**Additional file 2. Supplementary file. II: **Annotation of unigenes of *Erianthus arundinaceus, Saccharum spontaneum* and cultivated variety based on *Saccharum *sp as reference.

**Additional file 3. Supplementary file. III: **GO terms for DEGs of *Erianthus arundinaceus, Saccharum spontaneum *and cultivated variety.

**Additional file 4. Supplementary file. IV: **List of annotated DGEs with their FPKM for *Erianthus arundinaceus, Saccharum spontaneum* and cultivated variety.

**Additional file 5. Supplementary file. V: **List of novel DEG of *Erianthus arundinaceus, Saccharum spontaneum *and cultivated variety.

**Additional file 6. Supplementary file. VI:** Annotated differentially expressed AP2-ER-EBP, MYB related, WRKY and NAC transcription factor genes with FPKM values.

**Additional file 7. Supplementary File. VII: **Validation of expression of NAC genes through RT-PCR.

**Additional file 8. Supplementary File. VIII: **Validation of expression of representative genes through RT-PCR.

## Data Availability

All data from the presented study are included in this published article and supplementary information files. The transcriptome data generated in the present study were deposited in the NCBI Sequence Read Archive database under accession number PRJNA606674. https://www.ncbi.nlm.nih.gov/sra.

## Abbreviations

ROS: Reactive oxygen species
NCBI: National Center for Biotechnology Information
TF: Transcription factor
DGE: Differential gene expression
BLAST: Basic local alignment search tool
RT qPCR: Reverse transcriptase quantitative polymerase chain reaction
LP: Lipid peroxidation
CF: Chlorophyll fluorescence
CSI: Chlorophyll stability index
KEGG: Kyoto encyclopedia of genes and genomes
ICAR-SBI: Indian Council of Agricultural Research, Sugarcane Breeding Institute
NAC: N-acetyl cysteine
POX: Peroxidases
SOD: Superoxide dismutase
NGS: Next-generation sequencing
GO: Gene Ontology
